# Migration and education on social media: what migrants discuss about education in Facebook groups

**DOI:** 10.3389/fsoc.2023.1177411

**Published:** 2023-06-21

**Authors:** Helena Dedecek Gertz

**Affiliations:** Faculty of Education, University of Hamburg, Hamburg, Germany

**Keywords:** migrant online groups, latent ties, transnational education, Brazilian migration, Brazilians in Germany

## Abstract

Drawing from a networked perspective of migration and from the concept of “transnational education,” this paper investigates the education-related topics discussed in Facebook groups among Brazilian migrants in Germany. The paper examines the “latent ties” activated in migrant Facebook groups as part of networks that can be used to collect information about migratory pathways involving educational opportunities. A qualitative content analysis was conducted with 2.297 posts retrieved from six Facebook groups divided into location, vocational education and training (VET), and professional groups. The outcomes point out that there is a demand for transnational education beyond university degrees. Additionally, the paper highlights that latent ties can be used to collect and cross-check information in migratory contexts involving education.

## 1. Introduction

This paper investigates education-related information, opinions, and experiences shared by migrants and aspiring migrants on social media. While social media became an important part of migration journeys and education can be an important driver for some migrants, there has been relatively little investigation of the intersection of migration, social media, and education, except for a few studies (e.g., Chang et al., 2018; Jayadeva, 2020). Existing research on transnational education tends to focus on migrants pursuing university degrees abroad (Brooks and Waters, 2010; Beech, 2015) and the intersection of migration, media, and education also seems to go in that direction (Jayadeva, 2020). This paper broadens that scope to include other educational opportunities, such as vocational education and training (VET). Specifically, this paper aims to answer the research question: What education-related topics are discussed among migrants and aspiring migrants in social media groups? Empirically, it explores the case of Facebook groups of Brazilian migrants and aspiring migrants who wish to move to, or already live in, Germany. Henceforth, these groups are referred to as groups of Brazilians in Germany. The results illustrate how migrant networks of latent ties can be used to collect information about transnational education beyond university degrees.

In what follows, I provide contextual information to justify the focus on Brazilian migration to Germany. I also comment on the migratory conditions of nurses and information technology (IT) professionals to Germany, as these professional groups are relevantly present in the data. After that, I describe the concept of “transnational education” (UNESCO and Council of Europe, 2001) and how it is associated with social networks, particularly in migratory cases associated with educational levels beyond university studies (Carnicer, 2019; Fürstenau, 2019). I then present the concept of “latent ties” (Haythornthwaite, 2002) and describe how these are activated in migratory contexts, followed by a comment on Brazilian migrants' media use. In the research design section, I explain the sampling and data collection decisions and the procedures for conducting a qualitative content analysis (Schreier, 2012). Finally, I present the findings in four sections: the demand for transnational education beyond university degrees, the potential association of education-related topics with economic and gender differences, the perception that university studies can be sometimes irrelevant or associated with frustration in this migratory context, and the trust in information shared by latent ties in education-related topics. While it is not possible to assert whether people who exchanged information in the groups of Brazilians in Germany migrated and fulfilled their educational aspirations, the outcomes support that demand for transnational education goes beyond university degrees and that information shared in these groups is potentially relevant for migratory pathways associated with education.

## 2. Contextual information

In this section, I justify the focus on the migration of Brazilians to Germany and I outline the German labor market conditions for migrant nurses and IT professionals.

### 2.1. Brazilians in Germany and education

Although the number of Brazilians in Germany is small compared to migrants from other nationalities (Statistisches Bundesamt, 2022), Germany figures among the top five European countries of destination for Brazilian migrants (Ministério das Relações Exteriores, 2021). Therefore, from a Brazilian perspective, studying this population in Germany is of quantitative interest. From a German perspective, focusing on Brazilian migration provides insights into the migration of a socioeconomically diverse population who primarily migrate for reasons other than fleeing war and conflict. While the findings here are not generalizable, they might be similar among other populations, as social media use is widespread. The diversity of the Brazilian population is potentially reflected in the composition of Facebook groups of Brazilians in Germany, as almost 77% of that population uses social media (Statista, 2022) and such high figures seem to be prevalent regardless of class and gender, and across rural or urban areas (Spyer, 2018; IBGE, 2022; NapoleonCat, 2022). All sampled Facebook groups of Brazilians in Germany have over two thousand participants, which increases the likelihood of a diverse socioeconomic composition.

The Brazilian education system has historically disadvantaged people from low-income backgrounds, despite recent improvements such as the implementation of quotas in public universities (Câmara and Almeida, 2012; Sampaio and Oliveira, 2016; Windle, 2022). While there have been improvements in national education measurements, as of 2019, the average formal education attendance among Brazilians was only 9.4 years, with only 17% of people aged 25 or over having completed tertiary education (IBGE, 2022, p. 1). The need to work is the most common reason for Brazilians to drop out formal education, regardless of race or sex (IBGE, 2022, p. 11–13), what highlights the deficient policies for promoting educational continuity. Furthermore, structural racism is evident in the Brazilian educational system with the imbalance in tertiary education achievements. In 2017, only 31% of university graduates were Black or Brown, while 66% were white (Dias Silva, 2020, p. 23). Some of the students who cannot enter or conclude university degrees in Brazil see migration as a way to both access tertiary education and have an opportunity for upward mobility (Carnicer, 2019; Fürstenau, 2019).

In Germany, VET programmes are paid, last around 3 years, and are offered in a dual system. That means the students must attend teaching units and undergo supervised practical experience, similar to an internship. The starting salary for those beginning a VET programme in 2023 is at least 620 Euros with yearly increases thereafter (Bundesamt für Justiz, 2005). For aspiring nurses, the starting VET salary is around 1,166 Euros and, for an IT specialist in data and process analysis, the starting VET salary is 1,011 Euros (AUBI-plus, 2023). This salary is one of the conditions that enable young migrants from lower socioeconomic backgrounds who are excluded from the educational system in Brazil to pursue VET in Germany (Carnicer, 2019; Fürstenau, 2019). As of 2022, around 4.2 thousand Brazilians were enrolled in German universities (Destatis, 2022).

In Germany, the educational system tends to discriminate against migrant students, non-migrants with a migration history in the family, as well as non-migrants from low-socioeconomic backgrounds (Dumont et al., 2014; Olczyk et al., 2016; Mafaalani, 2021). This issue has been improving, however, it is still observable in the traditional selection patterns of the school system. After 4 years of primary education, students (aged 10) can be recommended to continue toward the *Gymnasium* (higher secondary education) or to other modalities that also allow access to tertiary education, but are seen as ‘less prestigious’. Pupils whose caretakers migrated to Germany tend to be discriminated against when it comes to recommendations to the *Gymnasium*, sometimes leading to a harder pathway in the access to tertiary education (Trebbels, 2015; Hunkler, 2016). For non-EU migrants, the path to VET in Germany is also a complex one: foreign school certificates are sometimes recognized as a lower level requiring migrants to repeat studies; apart from the formalities of the migration process, educational institutions require knowledge of German-language verified through expensive certificates which can make the way to VET no easier than the one to university.

### 2.2. Nurses and IT professionals

Two relevant professional groups in the dataset are nurses and IT professionals. In Brazil, nurses must hold a specific BA degree. IT professionals, on the other hand, do not need a mandatory higher education certificate to work in the field. In Germany, these two professions are listed as highly demanded on the official website dedicated to informing and attracting foreign professionals to the country (Make it in Germany, 2023).

Nurses' profession is regulated in Germany, hence, those who did not complete their training in the country are requested to have their certificates recognized, prove German knowledge through an officially recognized certificate, provide a certificate of mental and physical fitness signed by a German medical doctor, and show proof that they do not have a criminal record (Make it in Germany, 2022b). The costs of degree recognition vary according to federal states. In Hamburg, for instance, this may reach up to 722 Euros (Freie und Hansestadt Hamburg, 2022). Apart from those costs, applicants must also count with costs for, for instance, publicly sworn translations of their diploma, and German language exams, which can cost around 194 Euros in Brazil (Goethe-Institut, 2023). Based on all job announcements for nurses on the job-seeking portal *Glassdoor*, the average salary of these professionals in the private sector in Brazil is around 639 Euros monthly (Glassdoor, 2023d). For IT professionals working in Brazil, the mean salary is of around 2.380 Euros monthly (Glassdoor, 2023b). If a migrant has a job offer in Germany in an IT area with a salary of at least 52.560 Euros yearly, they can apply for a Blue Card visa. For that, they must show proof of at least 3 years of professional experience in the area, some professional certificate or examination, and a German language certificate, which may be disregarded in case the working language in their future company is not German (Make it in Germany, 2022a). In the private sector in Germany, the salary of an IT consultant can reach around 5.000 Euros monthly (Glassdoor, 2023c). Nurses in Germany receive around 3.100 Euros monthly (Glassdoor, 2023a).

In the global labor market, some trained nurses might perform domestic work after migration (Parreñas, 2015). To describe this phenomenon, Rhacel Parreñas introduced the concept of “contradictory class mobility” which, among other factors, arises from the devaluation of degrees obtained in the Global South (Parreñas, 2015, p. 118). Parreñas' interviewees expressed frustration about not being able to work in positions they were trained for in the Philippines, but also acknowledged that their migration allowed them to afford living standards they would not have been able to achieve otherwise. In other words, while their financial conditions improved, their perceived social status decreased (Parreñas, 2015, p. 219). Unlike health-related labor areas, the relatively de-regulated nature of the IT profession poses one barrier less for migrants working in that area. Still, migrant women working in IT seem to be disadvantaged, as requirements to live flexible and hypermobile lives “sit uneasily with gender norms, which demand women's participation in and taking responsibility for the running of geographically fixed households” (Raghuram, 2004, p. 174).

## 3. Transnational education and social networks

The term “transnational education” was proposed by the UNESCO to refer to “all types of higher education study programmes, or sets of courses of study, or educational services (including those of distance education) in which the learners are located in a country different from the one where the awarding institution is based” (UNESCO and Council of Europe, 2001, p. 2). Research in that area tends to focus on university education and, sometimes on private international schools (Adick, 2018). Among transnational education studies, the focus on university degrees comes along with an interest in institutional aspects (Waters, 2018), and migrants' expectations of acquiring cultural capital abroad (Waters, 2015; Yu, 2021). This kind of international mobility associated with education is held for a strategy of the middle classes, however, migrants from lower socioeconomic backgrounds also follow migratory projects based on educational aspirations and opportunities (Carnicer, 2019; Fürstenau, 2019). For some of these migrants, particularly in the German case, accessing VET is a means to fulfill their migratory goals and educational aspirations, as well as to support their families and achieve upward mobility (Carnicer and Fürstenau, 2021).

The migration of these Brazilian who wish to pursue VET in Germany supports the notion that migration is a project seldom planned and achieved individually, as migrants are embedded in “multi-stranded social relations that link together their societies of origin and settlement” (Basch et al., 2005, p. 8). This highlights the networked aspect of migration, where interpersonal connections are crucial for its accomplishment. Migrant network analyses have focused on strong and weak ties established through personal connections to describe migratory pathways involving education (Brooks and Waters, 2010; Beech, 2015; Carnicer and Fürstenau, 2019). These network connections can also be established online, and be used both to navigate dangerous migration journeys as well as to access information about university education abroad (Dekker et al., 2018; Jayadeva, 2020). Following that premise, the next section explores the establishment and uses of such online ties in migratory contexts.

### 3.1. Latent ties connections and information precarity in migration contexts

Social media have the potential to bridge different social groups: aspiring with settled migrants, job-seekers with job-holders, individuals with experience in the foreign educational system and newcomers (Komito, 2011; Nedelcu, 2012; Jayadeva, 2020; Kotyrlo, 2020). Such online connections bridged through social media can be named “latent ties,” i.e., connections “for which a connection is available technically but that has not yet been activated by social interaction” (Haythornthwaite, 2002, p. 385). On social media, latent ties can be the pool of users participating in an online forum: most of them do not know each other and a tie is established only when a user published something and receives replies from other users. These are at first rather instrumental connections that can serve to bridge people with experience in relevant matters for aspiring and newcomer migrants. For instance, latent ties can be activated in situations when official information is not available or when trustworthy information is hard to identify (Dekker et al., 2018). This situation has been described as “information precarity,” a condition of instability, identified particularly among asylum seekers using media to collect information for decision-making and to access personal data, which leaves them potentially vulnerable to misinformation (Wall et al., 2017; Dekker et al., 2018). While the migratory context presented in this paper is different from that of asylum seekers. These migrants seem to prefer social media information based on personal experiences or shared by users with whom a stronger social connection is already established, as these is perceived as more trustworthy (Dekker et al., 2018). Along with other platforms, Facebook groups are mentioned as convenient sources of information, however, the authenticity of such information is still cross-checked with personal acquaintances (Dekker et al., 2018, p. 8).

In migratory journeys related to education, Jayadeva (2020) has described the use of Facebook groups among Indian youth who wish to access universities in Germany. She suggests that information shared by latent ties can facilitate migrants' access to university education by expanding aspiring migrants' social capital (Jayadeva, 2020). In that context, Facebook groups are seen as a source of guidance in the process of applying to universities and even as a better alternative to paid education consultancy services. Although the migrant group studied by Jayadeva (2020) were in a different situation than the refugees studied by Wall et al. (2017) and Dekker et al. (2018), both groups of migrants rely on information shared on social media, evidencing the relevance of latent ties in migratory journeys, even in those in which information precarity is not necessarily the case (Jayadeva, 2020).

### 3.2. Brazilian migrants and latent ties

Brazilian migrants' fondness for social media is not new. Almost 10 years ago, Schrooten (2012) conducted a digital ethnography among Brazilian migrants living in Belgium who used Orkut (a platform deactivated in 2014). She pointed out that these groups “provide a variety of social capital … which assists in the migration transition” (Schrooten, 2012, p. 1,801). Schrooten reports political discussions, arrangements for in-person gatherings, and information exchanges about documentation and legal procedures and employment opportunities. Also researching Orkut, Oosterbaan (2010, 2013) reports that employment search was one of the most relevant topics in groups of Brazilians in Barcelona and Amsterdam. Oosterbaan (2013, p. 46) highlights that some groups support in-person meetings, which evidences a willingness to develop strong ties and trust in unknown people based on shared nationality, participation on an online forum, and possibly similar migratory situations. Latent ties are part of the networked aspects supporting migration aspirations and decision-making among Brazilian migrants (Dekker et al., 2016).

In the 2010s, both Oosterbaan (2010, 2013) and Schrooten (2012) reported similar levels of interaction in Brazilian Orkut groups in three European contexts, finding over a thousand communities with titles of “Brazilians in [location]” (Oosterbaan, 2010). Schrooten (2012) highlights that Orkut was embedded in migrants' daily life already before they migrated and, after migration, it became a relevant source of social, political, and cultural connection to Brazil. After Orkut was deactivated, migrants and aspiring migrants created new communities on Facebook—with no sign of diminished interest in social media networking. Researching Facebook groups of Brazilians in Sweden, Foletto (2018) reports groups with over 1.7 thousand participants and a mean of 66.8 monthly posts—similar amounts as among groups of Brazilians in Germany (see Table 1). The main feature of online migrant groups is, hence, information exchange and mutual support in the migration process. Latent ties established on social media are thus relevant among Brazilian migrants and aspiring migrants. It remains to be described the role of education-related topics in the activation of these ties.

**Table 1:** Interactions varied from over 800 words (in which concrete situations and doubts were explained, for instance) to 3 words (in short replies if someone asked for clarification to provide an accurate reply).

**Groups**	**Interactions**	**Words**	**Participants**
VET group 1	450	21.379	4.936
VET group 2	1,051	62.289	4.713
Location group 1	123	9.654	4.520
Location group 2	321	36.195	24.180
Nurses' group	153	13.909	3.944
IT group	199	11.789	2.480

In total, 2.297 interactions were collected. Although some groups had more interactions, the number of words is lower because the interactions (questions/answers) were shorter. For instance, Location group 2 had fewer interactions than VET group 1, but more words because interactions in Location group 2 were more detailed.

## 4. Research design

Education-related topics figure among the most prevalent ones debated in groups of Brazilian migrants in Germany (Dedecek Gertz and Süßer, 2022), hence these topics are among the most important cues to activate latent ties (Haythornthwaite, 2002). These education-related topics are connected to levels beyond university education, such as language learning and accessing VET (Dedecek Gertz and Süßer, 2022). This paper looks from a qualitative perspective at a share of these posts used to determine the prevalence of education-related topics in groups of Brazilians in Germany.

To select relevant groups, I first used Facebook's search tool to look for groups containing “Brazilian” and “Germany” (in Portuguese) and gained access to 43 “active groups,” i.e., groups with at least a thousand participants and three posts published in one week. Most groups required filling up a questionnaire upon entry, which I used to inform about my research interest. Also, on my Facebook profile, I am identified as a researcher and provide my contact information. These 43 active groups were divided into “location groups,” “professional groups,” “vocational education and training (VET) groups,” and “other” (Brazilians who wish to work as Au Pairs or to do voluntary work, “gardening for Brazilians” etc.). Anyone with a Facebook account could join the groups, however, due to the titles containing variations of “Brazilians in Germany,” the frequent discussion of topics related to regulations and requirements that apply to Brazilians in Germany, and the use of Brazilian Portuguese in the groups, the majority of participants are likely to be Brazilian.

After reading what was posted in these groups in 2020, some stood out due to the level of daily interactions: users engaged with posts commenting on their experiences, giving tips, or starting discussions based on their different experiences and opinions. Two groups from each relevant type were selected: two “location groups” and two “professional groups,” both with the highest number of interactions and the only two “VET groups” available. “Other” groups had no relevant educational-related interactions and were therefore not selected. The amount of collected interactions (questions and answers), words per group, and participants at the time of the data collection were as follows:

Posts made until the beginning of December 2020 were identified using Facebook's search tool and copied into a text editing document. Each group was screened for the following terms relating to formal education: learn, study, *Ausbildung* (the German word for VET), school, university, faculty, course, *Studienkolleg*,^1^ classes, and course. All posts and their respective comments containing at least one of these terms were collected. Although this selection encompasses a timeframe in which migration was limited due to COVID-19 restrictions, the levels of interactions were still high. In the dataset, the only mention of COVID-19 in association with education is a reply by a group participant informing that “students with a valid registration at a German university can enter the country” (Location group 2). The content of the posts also reveals that, regardless of the restrictions, this period was still used for planning migratory journeys: “even though there's not much to do now, I'm already collecting information” (VET group 1), writes a group participant.

After the data collection, posts that contained sensitive information, posts in which terms were used out of context, and posts containing advertising were eliminated. The posts were coded using MAXQDA according to the following deductive/inductive categories (Schreier, 2012).

- Educational levels being discussed
  ° Kindergarten
  ° School (*Gymnasium/Gesamtschule/Realschule/Hautpschule*)
  ° VET (divided by areas, e.g., care work, IT)
  ° University (BA, MA, Ph.D.)

- What was being discussed about education
  ° permanence in Germany (e.g., being enrolled in an educational institution to apply for a residence permit)
  ° professional aspiration (e.g., fulfilling requirements to apply for certain jobs)
  ° upward mobility (e.g., achieving a certain income)
  ° cultural capital (e.g., value attributed to diplomas)

- Questions
  ° recommendation or opportunity request (e.g., “I'm looking for a VET to work in a Kindergarten—if anyone knows about such an opportunity, let me know,” VET group 1)
  ° personal experiences (e.g., “has anyone undergone a certificate recognition lately and can tell me how it was?”, VET group 1)
  ° opinion request (e.g., “I'm having a hard time with the notary to have my high school certificate recognized—they claim a signature is missing but it is in fact there! What should I do?”, VET group 2)
  ° “information precarity” [“insecure, unstable, and undependable” information which could lead to potentially wrong, problematic or even dangerous decisions (Wall et al., 2017) such as “I heard say that having a university degree as a nurse is worthless. Is that true?”, Nurses' group]

- Answers
  ° based on “information precarity” (Wall et al., 2017) (wrong or incomplete information such as “people who cannot pay for VET can receive a public loan for that,” VET group 1)
  ° based on where to find official information (e.g., “you can find this information at the Job Center,” VET group 1)
  ° style or tone
    ▪ e.g., detailed information (e.g., a 100-word answer about how to register in the city hall and the documents one has to present to start VET, including a comparison between Brazilian and German documents, as found in VET group 1)
    ▪ e.g., frustrating, vague, or critical (e.g., “I'd also like more information about it but I could never find,” VET group 1, or “if you have no experience in the area and have no degree, you can forget it!”, IT group)
    ▪ e.g., supportive (e.g., “if you're an engineer you have a great advantage! Just go have your diploma recognized,” IT group)

A reliability test conducted by two trained coders resulted in a Cohen's Kappa value of 0.64, which can be considered “substantial” (McHugh, 2012, p. 279). To preserve users' anonymity, posts not will be reproduced word-by-word (Zimmer, 2010). As these groups can be found and accessed by anyone with a Facebook account, reproducing posts here would imply the possibility of de-anonymization. Thus, posts are paraphrased: names of places were substituted for similar ones, professions were substituted for other ones within the same field as the original, and the order of sentences was changed, as long as the sense of the content was not thereby lost. These paraphrased sentences serve as an illustration of patterns across the data.

## 5. Findings

Only two groups were bringing together Brazilians interested in pursuing higher education degrees in Germany: a group for Brazilian students and aspiring students of a university preparatory school (*Studienkolleg*) and one general group about studying in a German university. Although related to education, both groups did not reach the threshold to be considered “active groups,” thus they were not part of the dataset. This relative inactivity of Facebook groups for Brazilians who wish to study at a German university is contrasting with findings by Jayadeva (2020) about Indian aspiring migrants with that same goal. Nevertheless, the comparative absence of active groups of Brazilians wishing to access university education in Germany cannot be taken as evidence of lacking interest in that educational level. A hypothesis for that is that Brazilian students who wish to apply for university studies in Germany might rely on other sources of latent ties (such as existing specialized Instagram profiles or WhatsApp groups), on strong ties who already live in Germany, on paid consultancies, or ask questions directly to the institutions where they wish to study.

Although the active groups with the most participants is one of the location groups, the largest datasets came from the two VET groups. That is not surprising as VET groups are specific to those who are pursuing that educational level or who wish to do so, hence many interactions in these groups contained the words sampled in the data collection. Participants in location groups who ask questions relating to education are probably established in the city and are seeking educational options for their children, as school was a frequently used word in those groups. An explanation for the comparatively low educational-related topics in the IT group may be that these professionals can find jobs without a degree or without pursuing education in Germany. The number of interactions in the nurses' group is lower in comparison to the IT group, however, as the number of words in the nurses' groups is larger, interactions about education in the nurses' group are more extensive. This is probably due to the educational requirements for these professionals in Germany, which generates longer discussions.

As expected from forum-like online groups, interactions in the collected data follow a question-answers structure. Divergent personal experiences and opinions lead to debates but these were always replying to practical questions: no user posed divisive questions as a starting point, which suggests a common interest of participants to use the latent ties to exchange practical information and support each other (Jayadeva, 2020).

The findings indicate that there is a demand for educational levels beyond university degrees among migrants. Additionally, economic and gender differences become visible in discussions about expectations and frustrations related to education and migration. Also, university studies are discussed with frustration or are deemed irrelevant in the labor market, while it is associated with acquiring cultural capital for children. Finally, the detailed and supportive tone of some interactions, and the use of Facebook groups by some migrants to confirm information collected elsewhere point out that latent ties are trusted in education-related information exchanges.

### 5.1. Demand for transnational education beyond university degrees

Across groups, questions related to accessing university degrees are mostly associated with educational aspirations toward children or teenagers, as in:

“My daughter is almost finished with high school. After she graduates, we intend to move to Germany. I am looking for information about the process so that she can enter a German university in the future. I would like to know what would be the step-by-step application process, please.” (VET group 1)

“I like to travel a lot and I want my children to study at an internationally recognized German university. Although I earned more money in Brazil, I don't regret coming to Germany also because of that [traveling and educational aspiration toward children].” (IT group)

Latent ties are thus activated to collect information and reinforce the cultural capital attributed to university education in migratory trajectories (Waters, 2015; Yu, 2021). Another circumstance in which university education was mentioned was when migrants or aspiring migrants provided background information about themselves with the expectation of having accurate replies according to their case, hence a rather instrumental mention of that level of education:

“I already have a BA in Mathematics and Biology here in Brazil. Do you know if this could count toward diminishing the number of seminars I have to take for a VET?” (VET group 2)

“Can people who studied in a private university in Brazil also work in Germany?” (Nurses' group)

In location groups, university education is mentioned by newcomer students. However, the information exchange encompasses rather peripheral aspects and education is mentioned as a self-introduction:

“I'm a PhD student and I'm going to study in [city in Germany] in a few months from now. I'm trying to get to know the city from afar and learn how to find a place to stay. Which neighborhoods do you recommend?” (Location group 2)

The reasons why none of these groups seem to be used to collect information about accessing university education remains to be established, but the hypothesis I suggest for the lack of Facebook groups of Brazilians interested in accessing German universities might also apply to the lack of interactions about it.

VET requirements, experiences, and opportunities about that educational level, as well as school for children and teenagers, were main topics discussed across groups (cf. Dedecek Gertz and Süßer, 2022). In location groups, education inequalities in Brazil were also a topic of discussion. In one of these groups, when a newcomer migrant asked about which neighborhood would be recommended to live in, the availability of schools and Kindergarten was mentioned among other practical aspects, like transportation. Newly arrived parents also use the location groups to seek information about the educational system in Germany. Main concerns were the quality of educational institutions in Germany:

“I've heard that public education in Germany is great, and there are even people who think that it is better to study there than in the most expensive private school in Brazil. Yet, I've heard parents complaining because they cannot find a place in schools. Is it also difficult in the private ones?” (Location group 2)

These discussions further reveal a rather instrumental discussion around education, which might inform broader migratory and settlement decisions. Other school-related posts were about fees and experiences with children and teenagers' social and linguistic adaptation. Answers to such posts warned about the tendency of teachers not to recommend migrant children to the *Gymnasium*, consequently, leaving these children with a harder pathway to access universities (Trebbels, 2015; Hunkler, 2016):

“Schools will try to push many things on your son, but be sure to tell them you want him to go to the *Gymnasium*! They always try to push foreigners away from there. They'll tell you that the other school types are easier for your son and that he can still enter university after he has done VET—don't fall for that!” (Location group 2)

Although VET groups are not exclusively for care work or education-related professions, these areas are the most mentioned ones. In general, questions and answers about VET encompassed elementary ones, such as what it is, entry requirements, class schedules, teaching style, where to find vacancies, as well as the labor market:

“Could someone here explain to me what is *Ausbildung*?” (VET group 1)

“I've started a BA to be a nurse [in Brazil] but I didn't finish. Does anybody know if I can skip some classes from VET courses? And is it paid? I'm learning German on the level A2.” (Nurses' group)

“Did anyone in this group manage to pursue VET in an area that they reeeeally wanted? I see most people choosing care work because of the labor market…” (VET group 2)

“Can anyone tell me what are all the requirements to apply for VET. Documents and all? Thanks.” (VET group 2)

“Is the *Ausbildung* valid only in Germany?! Like, if I go back to Brazil this certificate is worth nothing?!” (VET group 2)

“I've graduated in nutritional sciences, but I've decided to change areas and now I'm specializing in web development here in Brazil. After that, I'd like to do a specialization in Germany. Is it a good idea? Does anyone recommend a VET course?” (IT group)

Questions like “what is an *Ausbildung*?” might be due to a translation issue or limited German knowledge because, in the Facebook groups, VET is mostly referred to with the German word which is probably still unkown to newcomer or aspiring migrants. Such questions about elementary aspects reveal that latent ties might be a first source of information about such educational levels beyond university degrees.

Some answers to questions about VET were lengthy and mentioned the need to show certificates of language proficiency and school completion, and some users also indicated where specialized information could be sought:

“You must have your documents and diplomas translated and recognized through the apostille convention, including high school, VET or higher education. After that, they will be forwarded to the Ministry of Education in the region where you live in Germany, where the equivalence will be done to determine whether or not you are eligible to pursue VET. In this process, the job center in your city can offer direct assistance. It is important to note that starting VET and learning the language are two separate things. To begin a VET course, you must have at least a B2 level of proficiency in the German language.” (VET group 1)

Questions about the labor market after VET also generated more elaborated replies, mostly based on personal experience:

“Much more important than having a VET certificate is to have experience. But even folks with less experience have proportionally higher salaries here because of the high demand for IT professionals, I know this because we're hiring a lot in the company I work for and I am participating in the selection processes.” (IT group)

The discussion about educational levels beyond university degrees appears to be an important one across the sampled groups. Education seems to be mostly discussed on an instrumental level: as a means to fulfill migratory aspirations by being able to apply for a visa as well as to make decisions such as where to live based on access to schools for children. Accessing VET, in particular, is viewed as a way to fulfill both educational and migratory aspirations (Carnicer, 2019; Fürstenau, 2019), even if that might result in frustration for a perceived devaluation of skills or having to choose a profession based mostly on the labor market (Parreñas, 2015).

### 5.2. Economic and gender differences in education-related discussions

Economic and gender disparities are particularly visible in the groups of IT professionals and nurses. These professions are highly gendered, and although it cannot be generalized that mostly men participate in the IT group and mostly women participate in the nurses' group, in the dataset, most posts in the nurses' group were made by users with typical Brazilian female first names, who used adjectives and pronouns in the female form. In the IT group, posts were mostly made by users with typical male first names, who used adjectives and pronouns in the masculine form. Based on the composition of the dataset, it appears that education-related topics are mostly discussed by women in the nurses' group, and by men in the IT group, even though the overall gender balance in both groups cannot be determined with absolute certainty.

In the nurses' group, common debates were about whether the salary paid in Germany is enough to cover basic expenses, including children's education: they wish to know about costs and experiences with German schools, the chances migrant children have of being accepted at a German university or VET:

“It was a very difficult period because I came first to Germany and my daughter and husband afterwards. But I see how much this pain was worth it, especially for my daughter. Here children can have the profession they want, independently of the family's financial situation.” (Nurses' group)

“Can I live in the city of Hildesheim and work as a nurse on a salary of 1.200 Euros during VET? I'm wondering about the cost of living for my family…” (Nurses' group)

“Living in Germany will not make you rich, but you'll have a better life, better education for your children, more opportunities and safety! Here you can earn a good or a bad wage, but NEVER as bad as in Brazil.” (Nurses' group)

Children are also mentioned in connection to hiring companies that offer services to mediate nurses' migration—such companies are one of the most discussed topics in the group. Some posts expose frustration and regrets, as a comment expressing concerns that such companies would not accept candidates with children. Some of these hiring companies pay for German courses and for VET in Germany, which is mentioned as an attractive offer, especially for aspiring migrants from low socioeconomic backgrounds, yet this process is not always transparent and reliable (CORRECTIV, 2020). Apart from being able to present the expected educational certificates, a central topic of concern regarding education and profession in the nurses' group is language knowledge. Aspiring migrants who are probably in their first steps of finding ways to migrate are mocked when asking about the need to have a good command of the German language: with sarcasm, other group participants tell the newcomer that it is “impossible” to acquire the needed certification and to work in Germany as a nurse without knowing German. Although participants show support and understanding for aspiring migrants, criticism is sometimes present in the nurses' group:

“I came to Germany through that company. I am at the B2 level and these months have been very intense with German classes from Monday to Friday. Class attendance is very important, because not only the school evaluates your performance but also your future employer. The point is, you need to learn German, there is no magic, you need to dedicate yourself and make an effort to understand the language.” (Nurses' group)

“Do you even understand German? Have you read the whole explanation about degree validation on the website? Read it first and only then come discuss this with me.” (Nurses' group).

In the IT group, there are mentions of salary expectations higher than in the nurses' group. However, these comments are contradicted by a few other group participants demanding aspiring migrants who are IT professionals to be careful not to assume that a five-digit income is a norm in the German job market:

“A high salary for me is above 97k. I don't know any IT professional who has a hard time in Germany, even if they speak only English. I spent the first 10 years of my career in Brazil and nowadays I find it funny that I thought my salary was good there. Here I buy what I want and I can still save some money.” (IT group)

“At the beginning, you'll earn more than 40k but with some years of experience you'll earn up to 100k in some regions in Germany.” (IT group)

“I already earn over 5 thousand Euros in Brazil. I'm afraid my quality of life living with that amount in Germany would fall.” (IT group)

Income expectations appear twice in relation to children's education: once in a discussion about the “advantages of public services in Germany” and another in a post by a user expressing the wish that their children will access “internationally prestigious German universities” (mentioned in Section 5.1). The state's financial support for parents is also positively associated with migrating with children. Learning German to access the job market and pursuing educational aspirations in Germany is associated with maintaining a middle-class status or with upward mobility.

“With my Portuguese passport, will I be able to work and bring my wife with me? I'm thinking about waiting until my son is 6 and then go to Germany for a master's degree. Do you think it would be worth it?” (IT group)

Answering the question above: “Bro, why would you miss years of child benefits?! Come soon, dude!” (IT group)

High salaries, no need to prove formal education or language knowledge, EU citizenship,^2^ and migrating without having to rely on intermediary companies reveal a comfortable status that IT workers possibly already had in Brazil and are likely to maintain in Germany. Due to high salaries and the disregard for state-recognized degrees in hiring processes, migrants working in IT might have an easier migratory pathway if compared to nurses (cf. Raghuram, 2004). The shared interest in migrating and maintaining socioeconomic status working in IT seems to create room for fraternal conversations in this group, in which unknown people treat each other as “bro” and “dude”. Whereas, in the nurses' group, the lack of German knowledge was sometimes mocked, in the IT professionals' group this was a neutral fact: if a person speaks German, good for her, if not, group participants tended to still encourage her to pursue their plans. Such supportive messages might contribute to building a community sense among people who serve as latent ties (Jayadeva, 2020) and contribute to ascribing trustworthiness to information shared by participants in these networks, as further discussed in Section 5.4 (“trust in latent ties in education-related topics”). The frustration expressed in the nurses' group might also serve as a way to build a sense of shared experience among group participants, which might fulfill a similar role as supportive answers among IT professionals. The economic and gendered differences in connection to education are evidenced in how group participants exchange information about children's education and how that connects with other migratory conditions, such as salary expectations and educational requirements for each profession. In both groups, migrating with children raises concerns about education and this is discussed rather in terms of maintaining or achieving a class status (Waters, 2015), however differences based on apparent previous socioeconomic conditions become visible in the ways how that is discussed: while IT professionals hope their children will access “prestigious universities,” nurses hope for better education and opportunities for their children.

### 5.3. University studies: “irrelevance” and frustration

In Brazil, nurses need a bachelor's degree to exercise their profession. In Germany, they need a VET certificate, which must be recognized by the German state through a national exam. Particularly in the nurses' group, this classification of university degrees as VET is debated with frustration:

“In Germany, our degrees are recognized as VET, which is not even considered higher education here.” (Nurses' group)

“I've been a nurse for 20 years in Brazil. I received a letter a few days ago informing me that I must do a training which lasts from 3 to 6 months to have my degree recognized. The more I learn about how things work here, the more confused I get.” (Nurses' group)

“My brother works at the university hospital here, even though he is very experienced, he can't do half of what he did in Brazil. Although he arrived here with a reasonable level of German, he had to do very simple work. He was frustrated when he saw the other nurses doing crap procedures and he couldn't say a word.” (Nurses' group)

“I have a BA in pedagogy but I was just informed that it won't be recognized here as such. Has anyone been through this as well? What should I do?” (VET group 1)

Answering the question above: “If they told you that, that's it. You'll have to study again here and you can spare your energies fighting this system because in Germany it's like that.” (VET group 1)

Having work experience disregarded and university degrees recognized as VET in Germany is met with remorse, particularly among nurses. The high value attributed to university degrees in the nurses' group is also visible in the comments of a group participant who challenged others by claiming to hold a bachelor's in the area from a German university. This participant felt offended by those who denied the existence of that degree. Such attitude illustrates the social value attributed to the degree among these professionals and the frustration of not having it recognized or compensated as that of a university graduate in Germany.

For IT professionals, due to the deregulated nature of this labor market, having a degree is discussed as being an asset but not particularly relevant:

“I have friends who were also hired in Germany [for an IT position] and don't have any degree or certificate, but of course that makes it easier to find a job.” (IT group)

“I know several people without a degree who work with me, even earning more than me. Many started a degree, but didn't finish it. It's quite irrelevant.” (IT group)

Unlike in the nurses' group, in the IT group, there were fewer group participants interested in pursuing VET. This might be because Brazilian degrees or VET certificates are accepted by companies in this profession without the need of having them officially recognized or because of the promises of finding such jobs by proving experience in the area, as mentioned in the group.

Facebook groups are also used by aspiring migrants with university degrees frustrated with salaries in Brazil and interested in VET as a means to migrate:

“I need help. I have a MA degree in pedagogy and I earn way too little [in Brazil]. I want to try to go to Germany, so I think pursuing VET is the best way. I don't want to go to university in Germany! I want something that gives me a job.” (VET group 2)

“I have a BA in translation, does anyone know if with that I can work at a Kindergarten? I'm fine even to be an assistant or something like that.” (Location group 2)

Within the group of IT professionals, having university degrees is described as beneficial, but not essential for finding a job in Germany. On the other hand, in the nurses' group, a topic associated with university studies is frustration due to the perceived devaluation of their degrees in Germany. Similarly, other aspiring migrants with university degrees in other areas display frustration, as they perceive their degrees as being undervalued in Germany. These two latter cases seem aligned with Parreñas (2015)' concept of “contradictory class mobility”: migrants seem to perceive, or even aspire to, a decrease in social status associated with university degrees and accept jobs for which they may be overqualified, in exchange for an expected economic improvement and stability as well as gaining access to public services, such as schools for their children.

### 5.4. Trust in latent ties in education-related topics

Although not always the case, interactions across groups were mostly supportive and group participants provided suggestions and were willing to share their experiences. That is particularly the case of users searching for information about the requirements to pursue VET, which is also linked to the requirements to be granted a visa. Some participants mention that they have already searched for information in official sources, such as educational institutions and governmental websites, yet they claim to be confused and seek clarifications and ask for personal experiences on Facebook groups. Users who claim to have a good command of German also mention having difficulty finding information and thus resort to Facebook groups:

“I've looked around in the websites and it looks like there is no English-taught BA in Engineering here. But maybe there are for other areas? Does anyone know of any degree like that?” (Location group 2)

“To work in Germany in a Kindergarten, what should I do? I've graduated in Linguistics [in a Brazilian public university] and I already speak German.” (VET group 1)

Even elementary questions, such as “what is an *Ausbildung*?”, receive answers such as:

“Unlike Brazil, where hairdressers and bricklayers learn their trade on the job, almost all professions in Germany require VET, which is called *Ausbildung*. It is a very good way to enter the job market because students are referred to work even during the course and most of the time they get a real job after they're done studying. It's also a good way to afford costs of living because you get paid to do an *Ausbildung*.” (VET group 1)

The author of this last reply draws a comparison between Brazil and Germany, explains what is an *Ausbildung*, and what are its advantages, formulating an elaborated answer. Such an answer could have been found on other websites (the Goethe Institute in Brazil or blogs of Brazilians living in Germany, for instance), however, the user posing the question prefers to activate latent ties to find an answer. Some of these answers are accompanied by motivational messages along the lines of “I have done it, you can do it too” or “believe in yourself and go get it” (VET group 1). Asking elementary questions, and receiving elaborated and supportive answers for it points out both that some aspiring migrants might use Facebook groups as their first source of information about education beyond university levels in Germany and that group participants share a sense of empathy or solidarity, similarly as described by Jayadeva (2020).

Another use of these groups seems to confirm whether the information collected elsewhere is accurate. Such questions usually do not specify the source of the information the person holds:

“I hear some people saying that [an institution] offers payment to pursue VET. I'd like to know whether that's true. Does anyone have experience with this?” (VET group 2)

“I've heard that there is some system for migrants to complete high school here. Do you think I could do that with my elementary school certificate?” (VET group 2)

It appears that these individuals are seeking second opinions for their hearsay information by consulting with unknown participants of Facebook groups. Although they may also seek second opinions on official websites or by contacting institutions, relying on latent ties to clarify doubts demonstrates that the experiences and opinions of Facebook group members about education in Germany are considered valuable and trustworthy. Finally, some group members empathize with the position of these aspiring or newly arrived migrants who are seeking information on Facebook groups and provide detailed and supportive answers. This might reinforce a perception of reliability or trustworthiness among the group. Dekker et al. (2018) described a similar phenomenon, but in the other direction, with migrants checking information collected from latent ties against the knowledge and experience of personal acquaintances. However, in the case of migrants who are not fleeing war or conflict and who employ social media to search for education-related information, latent ties appear to be relied upon and utilized for verifying information.

## 6. Conclusion

Non-university education is a main topic of discussion in the analyzed migrant Facebook groups (Dedecek Gertz and Süßer, 2022). University studies were also present in the interactions, however other educational levels, VET in particular, were an important demand. This outcome is consistent with other studies which point out that transnational education goes beyond university degrees and extends to educational levels that may facilitate access to education abroad for students from lower socioeconomic backgrounds (Carnicer, 2019; Fürstenau, 2019). These other studies relied on network analyses of strong tie connections to describe how these migrants accessed education abroad. While it is not possible to establish whether the aspiring migrants whose interactions on Facebook groups were described here indeed migrated, it is possible to claim that latent ties (Haythornthwaite, 2002) are used to acquire and cross-check information about education abroad, particularly VET. Hence, such ties established online are also part of networks of transnational migratory pathways involving education (Jayadeva, 2020)—also beyond university levels.

Education is often discussed on an instrumental level among aspiring migrants in nurses' and VET groups, with a focus on migrating through such educational opportunities. In location and IT groups the interest in education may assume a character of cultural capital, especially when it comes to children's education. The interactions reveal concerns about fees in international schools, about the possibility of children not being able to reach the *Gymnasium* and proceed to a “prestigious German university,” a similar concern of East Asian families of the middle and upper-middle classes who expect that their children would acquire cultural capital through transnational education (Waters, 2015). Other aspects of formal education, such as proof of language proficiency and degrees, are relevant for all groups, but not much to IT professionals, who comment on the possibilities of applying for jobs and visas without either knowing the language or holding tertiary education degrees. This is unthinkable for migrant nurses, who must master the language and either have their Brazilian bachelor's degree recognized in Germany or pursue VET in the country, which reveals the advantages of tech workers, who may face fewer hindrances and frustrations than care workers (cf. Raghuram, 2004). Hence, instead of using education as a means or a goal of migrating, migrants with more stable economic means seem to associate education with the maintenance of socioeconomic status. For aspiring migrants in other economic conditions, a contradictory class mobility (Parreñas, 2015) by pursuing VET seem to be desirable and sometimes recommended by other migrants in the groups, even if the devaluation of work experience and Brazilian university degrees is met with frustration.

Differently than in other migratory contexts, when information collected online is validated with strong or weak ties (Dekker et al., 2018), some of the interactions described here point out that latent ties are also used to confirm information collected elsewhere. This is most likely related to the different contexts of migrants—who are not fleeing war and requesting asylum, as the ones researched by Wall et al. (2017) and Dekker et al. (2018)—and the sampling for education-related topics, as migration associated with education demands a preliminary personal organization, having documents translated, providing language certificates, among other requirements. The supportive tone, attentive answers, and descriptions of personal experiences provided by some group participants might also contribute to the apparent trust in information shared by latent ties. This interest in seeking information and support among migrants in similar situations aligns with what was observed a decade ago, on a social media platform which no longer exists, among Brazilian migrants (Schrooten, 2012; Oosterbaan, 2013), and also more recently, among Indian aspiring migrants on Facebook and WhatsApp groups (Jayadeva, 2020). Hence, this phenomenon might also be identified among migrants from other nationalities and it is likely to continue regardless if a certain social media platform ceases to exist.

Some of the information shared in these groups is not accurate and the snapshot of the experience of someone else might convey only a partial truth about migrating and accessing education abroad. Hence, the role of information precarity (Wall et al., 2017; Dekker et al., 2018) in the context of migration involving education can be further researched through interviews. Another aspect that demands further research is whether education generally and VET, more specifically, is also an important topic in other national contexts. Because in Germany VET studies are paid, education-related debates are likely to be different among migrants seeking to access education in other countries. The economic and gender differences identified particularly in groups of nurses and IT professionals raise the question of whether latent ties connections may reproduce inequalities in education-related migratory pathways similarly as identified in networks of strong and weak ties (Beech, 2015). Future research could further examine whether latent tie connections contribute to or challenge these inequalities.

## Data availability statement

The dataset used for this article is not available for immediate access due to concerns about potential de-anonymization.

## Ethics statement

Ethical review and approval was not required for the study on human participants in accordance with the local legislation and institutional requirements. Written informed consent for participation was not required for this study in accordance with the national legislation and the institutional requirements.

## Author contributions

The author confirms being the sole contributor of this work and has approved it for publication.

## Footnotes

^1^Public educational institutions that offer a one-year preparatory course for students who finished school outside Germany and wish to access a German university.

^2^Some EU-countries allow double citizenship based on the principle of jus sanguinis, i.e., for the case in this paper, Brazilians who can prove they have ancestors from, for instance, Germany, Italy, Portugal or Spain (the most common EU-ancestries in Brazil) can apply for citizenship in those countries.
